# Correction: Induced pluripotent stem cell-derived monocytic cell lines from a NOMID patient serve as a screening platform for modulating NLRP3 inflammasome activity

**DOI:** 10.1371/journal.pone.0249807

**Published:** 2021-04-05

**Authors:** Ryosuke Seki, Akira Ohta, Akira Niwa, Yoshinori Sugimine, Haruna Naito, Tatsutoshi Nakahata, Megumu K. Saito

The affiliation for the third author is incorrect. Akira Niwa is not affiliated with #2 but with #1: Department of Clinical Application, Center for iPS Cell Research and Application (CiRA), Kyoto University, Kyoto, Japan.

## References

[1] Seki R, Ohta A, Niwa A, Sugimine Y, Naito H, Nakahata T, et al. (2020) Induced pluripotent stem cell-derived monocytic cell lines from a NOMID patient serve as a screening platform for modulating NLRP3 inflammasome activity. PLoS ONE 15(8): e0237030. 10.1371/journal.pone.0237030 32810141 PMC7437452

