# Supplementary figures and images for: Induced pluripotent stem cell-derived monocytic cell lines from a NOMID patient serve as a screening platform for modulating NLRP3 inflammasome activity
Source: PLoS One. 2020 Aug 18;15(8):e0237030. doi: 10.1371/journal.pone.0237030 (PMC7437452; doi:10.1371/journal.pone.0237030)

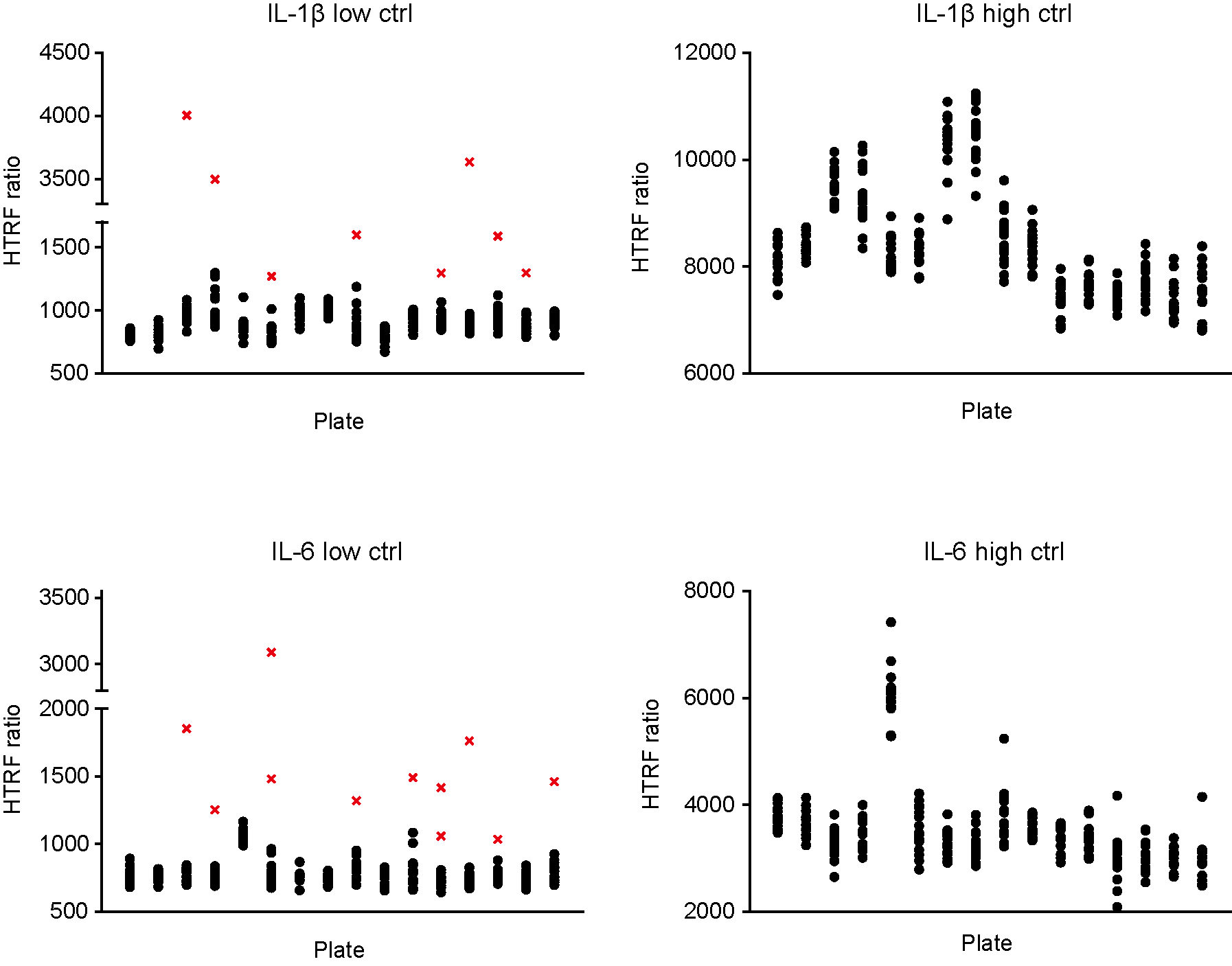

Supplement: S1 Fig — Each of the 16 plates contained 16 high (stimulated) and 16 low (not stimulated) controls treated with DMSO. Values falling outside of the mean ± 3SD were excluded as outliers (red crosses) except when Z’-factors and S/B ratios were calculated. (TIF) [file pone.0237030.s001.tif]

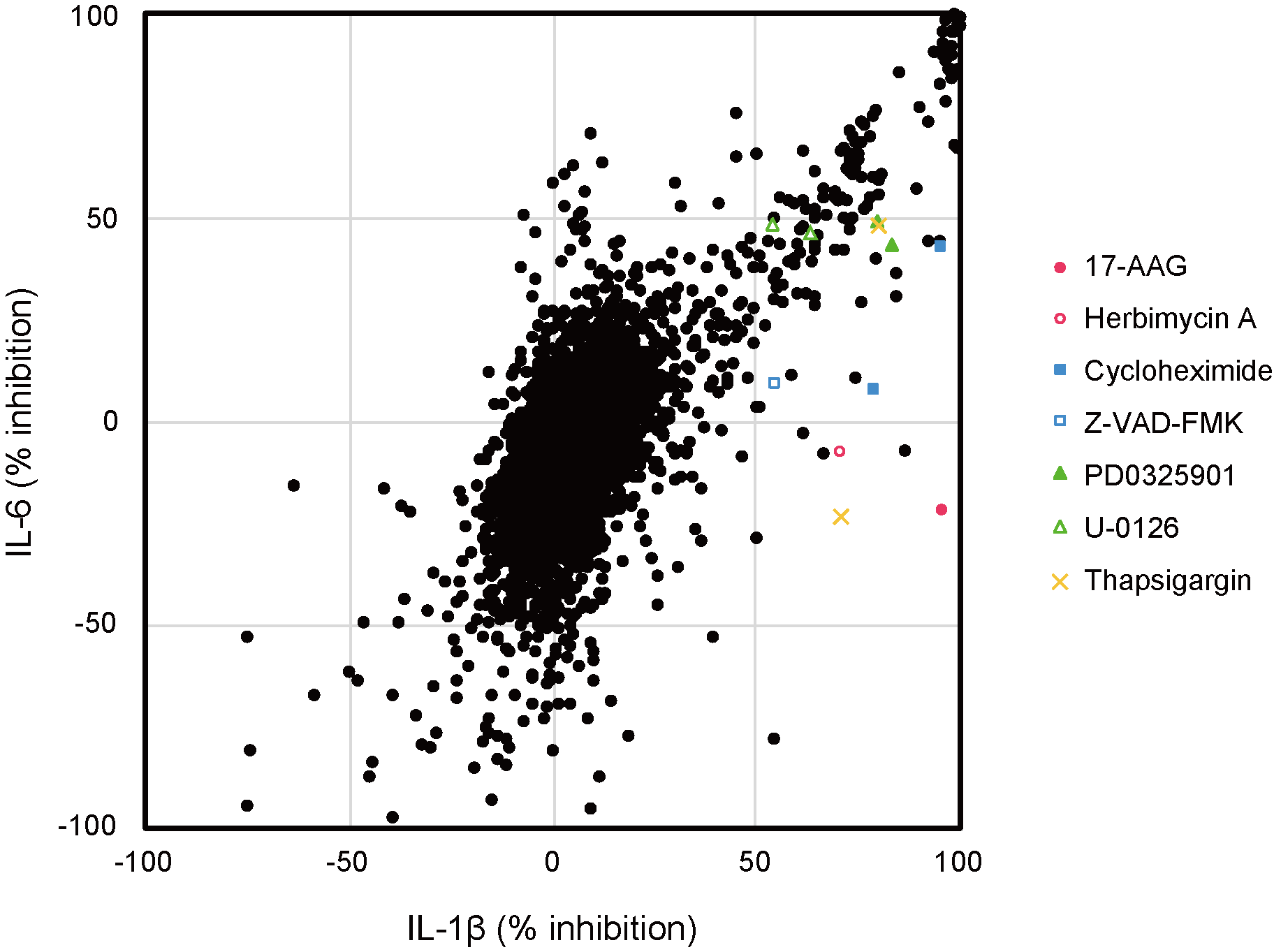

Supplement: S2 Fig — Percent inhibitions for IL-1 (x-axis) and IL-6 (y-axis) within a range of -100 to 100 are shown. Seven hit compounds are marked. (TIF) [file pone.0237030.s002.tif]

Fig 2c

Positive controls

MY

B

MD

iPS-MLs

MY

B

MD

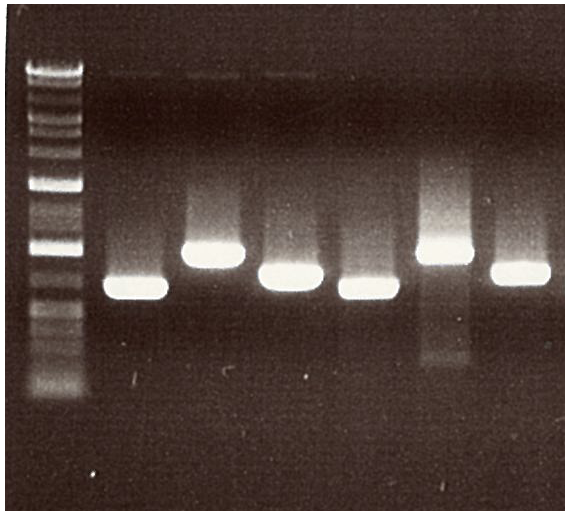

Supplement: S1 Raw image — (PDF) [file pone.0237030.s005.pdf]
