# Supplementary material for: Induced pluripotent stem cell-derived monocytic cell lines from a NOMID patient serve as a screening platform for modulating NLRP3 inflammasome activity
Source: PLoS One. 2020 Aug 18;15(8):e0237030. doi: 10.1371/journal.pone.0237030 (PMC7437452; doi:10.1371/journal.pone.0237030)
Supplement: S1 Table — (PDF) [file pone.0237030.s003.pdf]

Banding technique	Q-banding
Total counted cells	20
Total analyzed cells	20
Total karyotyped cells	8
Karyotype	46,XY,1qh+,9qh+[6]/<br>46,XY,1qh+,chrb(4)(q23),9qh+[1]/<br>46,XY,1qh+,dup(1)(q25?q43?),del(4)(q25?),9qh+[1]
